# Performance Comparison of Different Neuroimaging Methods for Predicting Upper Limb Motor Outcomes in Patients after Stroke

**DOI:** 10.1155/2022/4203698

**Published:** 2022-06-06

**Authors:** Jingyan Tao, Zhaoqing Li, Yang Liu, Jianhua Li, Ruiliang Bai

**Affiliations:** ^1^Department of Physical and Rehabilitation Medicine, Sir Run Run Shaw Hospital, School of Medicine, Zhejiang University, Hangzhou 310029, China; ^2^Department of Physical and Rehabilitation Medicine of Sir Run Run Shaw Hospital and Interdisciplinary Institute of Neuroscience and Technology, School of Medicine, Zhejiang University, Hangzhou 310029, China; ^3^Key Laboratory of Biomedical Engineering of Ministry of Education, College of Biomedical Engineering and Instrument Science, Zhejiang University, Hangzhou 310029, China; ^4^MOE Frontier Science Center for Brain Science and Brain-Machine Integration, School of Brain Science and Brain Medicine, Zhejiang University, Hangzhou 310029, China

## Abstract

Several neuroimaging methods have been proposed to assess the integrity of the corticospinal tract (CST) for predicting recovery of motor function after stroke, including conventional structural magnetic resonance imaging (sMRI) and diffusion tensor imaging (DTI). In this study, we aimed to compare the predicative performance of these methods using different neuroimaging modalities and optimize the prediction protocol for upper limb motor function after stroke in a clinical environment. We assessed 28 first-ever stroke patients with upper limb motor impairment. We used the upper extremity module of the Fugl-Meyer assessment (UE-FM) within 1 month of onset (baseline) and again 3 months poststroke. sMRI (T1- and T2-based) was used to measure CST-weighted lesion load (CST-wLL), and DTI was used to measure the fractional anisotropy asymmetry index (FAAI) and the ratio of fractional anisotropy (rFA). The CST-wLL within 1 month poststroke was closely correlated with upper limb motor outcomes and recovery potential. CST‐wLL ≥ 2.068 cc indicated serious CST damage and a poor outcome (100%). CST‐wLL < 1.799 cc was correlated with a considerable rate (>70%) of upper limb motor function recovery. CST-wLL showed a comparable area under the curve (AUC) to that of the CST-FAAI (*p* = 0.71). Inclusion of extra-CST-FAAI did not significantly increase the AUC (*p* = 0.58). Our findings suggest that sMRI-derived CST-wLL is a precise predictor of upper limb motor outcomes 3 months poststroke. We recommend this parameter as a predictive imaging biomarker for classifying patients' recovery prognosis in clinical practice. Conversely, including DTI appeared to induce no significant benefits.

## 1. Introduction

Stroke is a major disease that can lead to disability. Upper limb motor impairment is common after a stroke and may compromise patients' quality of life and severely affect their daily living [1, 2]. Predicting relevant upper limb motor outcomes and recovery potential is challenging for rehabilitation therapists and clinicians. Previous studies have explored several clinical scales and imaging techniques to identify the relevant predictors for motor recovery after stroke. Early studies predicted motor outcome by clinically assessing initial motor dysfunction [3]. Wegen et al. [4] proposed that two simple movements, shoulder abduction and finger extension, within 72 hours after stroke could predict recovery of hemiplegic upper limb function at 6 months. Increasing studies have tried to predict motor outcomes by neuroimaging markers measuring the structural integrity of the CST or the excitability of the motor cortex. For example, task-related brain activation in functional magnetic resonance imaging (fMRI) was correlated with hand function recovery [5]. fMRI activation in the supplementary motor area obtained early after stroke provided independent prediction of long-term motor outcome [6]. Several studies have predicted recovery based on brain structural MRI (sMRI) or diffusion tensor imaging (DTI) [7–11].

Early conventional sMRI (e.g., T1- or T2-based MRI) showed that lesion size was correlated with motor dysfunction [7, 8, 12]. DTI-based studies further indicated that the lesion location, especially those involving critical structures such as motion-related cortical areas (primary and nonprimary motor areas), the corona radiata, the posterior limb of the internal capsule (PLIC) [13], and the CST, could predict upper limb function recovery potential [9–11]. DTI-derived metrics of the CST, specifically the fractional anisotropy asymmetry index (FAAI) and the ratio of fractional anisotropy (rFA) between ipsi- and contralesional CST, are the most frequently used predictor variables in prognostic studies [14, 15]. Some studies quantified lesion size and location as the concept of lesion load—a combined measure of the stroke lesion overlapped with a canonical CST [16, 17]. Feng et al. [16] reported that CST-wLL in the acute phase was a strong predictor of upper limb motor recovery at 3 months.

Structural imaging analysis is usually based on DTI. However, owing to its costs and hardware requirements, DTI is not routinely performed in stroke and rehabilitation units, especially in developing countries. T1- and T2-weighted images are involved in early MRI scans after strokes. Few studies have compared the performance of sMRI-derived CST-wLL and DTI-derived metrics for predicting recovery of upper limb motor function. In this study, we aimed to optimize the clinical prediction model protocol in the clinical environment by comparing the performances of different neuroimaging modalities. We focused on whether including DTI would significantly benefit patients. Thus, we quantitatively analyzed the CST-wLL, which is calculated from the lesion volume in the T2 image overlaid on the CST map from the standard template. Further, we explored the relationship between the CST-wLL and upper limb motor function after stroke and compared them with other DTI-derived predictor variables. We hypothesized that compared with DTI-derived metrics, CST-wLL can more precisely predict upper limb motor recovery after stroke.

## 2. Methods

### 2.1. Study Participants

**Figure 1:**
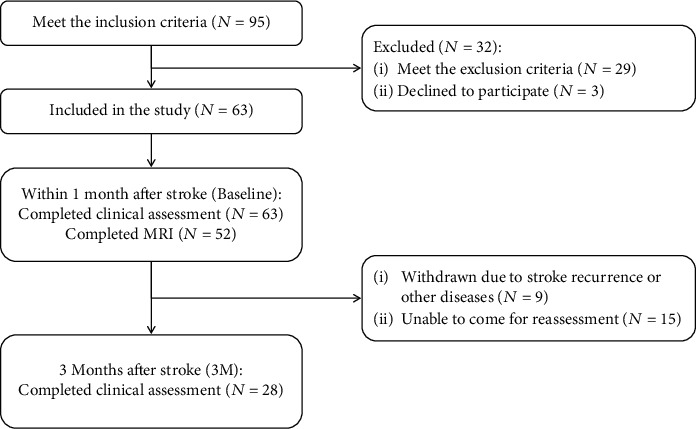
Flowchart of the recruitment process.

This prospective study included patients with first-onset stroke exhibiting varying degrees of unilateral limb motor impairment. Clinical and neuroimaging assessments were performed within 1 month after the stroke (baseline), and motor function recovery was followed up at least 3 months after the stroke. All participants were inpatients at the Department of Physical and Rehabilitation Medicine, Sir Run Run Shaw Hospital, School of Medicine, Zhejiang University, between 2017 and 2021, who underwent 1-month inpatient rehabilitation treatment including standard physical therapy and occupational therapy (Figure 1). The clinical trial was conducted in accordance with the Helsinki Declaration after approval by the Ethics Committee of the Sir Run Run Shaw Hospital and Zhejiang University School of Medicine. All relevant procedures were conducted with patients' full understanding and receipt of their written consent. Inclusion criteria were (1) cerebral hemisphere infarction (ischemic) confirmed via routine MRI scanning, (2) first-onset stroke, (3) hemiplegia of one limb within 1 month after onset, (4) stroke subtype based on TOAST criteria: large vessel atherosclerotic disease, and (5) age > 18 years. Exclusion criteria were (1) infarcts in both cerebral hemispheres, (2) cerebral hemorrhage or hemorrhage after stroke, (3) disturbance of consciousness, (4) unstable vital signs or failure of vital organs, (5) inability to remain in a supine position for 20 minutes, (6) MRI contraindications, (7) previous history of other neurological or orthopedic diseases that may have affected upper limb function, and (8) history of severe dementia or depression not controlled by medication. Patients were reassessed 3 months after their stroke.

### 2.2. Clinical Measures

The upper extremity (UE) module of the Fugl-Meyer assessment (UE-FM) was conducted within the baseline period (within 1 month of onset) and 3 months after the stroke. The UE-FM scale contains 33 items to comprehensively quantify upper limb motor impairment. The therapist, who was blinded to the imaging results, observed 30 voluntary UE motions and 3 tendon tap responses and provided an ordinal rating (2 = approximate to normal ability/response, 1 = partial ability, and 0 = unable to perform/no response). The scores were added to obtain a total score and recorded (66 maximum). Higher scores indicated less limb impairment; lower scores indicated more limb impairment. The scale has excellent intrarun and interrun reliability, test-retest reliability, and internal consistency [18].

### 2.3. Image Processing and Data Analysis

The GE Discovery 750 W 3.0 THD dual-gradient 16-channel MRI system with an 8-channel head and neck combined coil was used for the MRI. T1-weighted high-resolution imaging, T2 fluid-attenuated inversion recovery (FLAIR) imaging, and DTI were performed. Structural T1-weighted images were obtained using fast gradient echo sequencing prepared via three-dimensional (3D) magnetization (repetition time: 8.5 ms; echo time: 3.9 ms; 150 slices; voxel size: 1 × 1 × 1 mm^3^). Additional neuroimaging sequencing parameters were T2 FLAIR (repetition time: 11000 ms; echo time: 125 ms; 31 slices; voxel size: 0.49 × 0.49 × 6.50 mm^3^) and DTI (32 directions; *b*-value: 1000 seconds/mm^2^; 60 slices; voxel size: 1.75 × 1.75 × 1.75 mm^3^, TR = 8000 ms, TE = 80.7 ms). Neuroimaging data were analyzed by radiologists who were blinded to all clinical data.

### 2.4. Calculation of CST-wLL

Images were preprocessed with FSL 5.0.9 (https://fsl.fmrib.ox.ac.uk/fsl/) [19]. Lesion areas were manually drawn on the T2 image, which was linearly matched with the respective T1-weighted image and then transformed to the lesion mask. The CST-wLL was calculated using MATLAB with homemade script as previously described [17]. In contrast to Lin et al. [17], the canonical CST used in this study was determined by nonlinearly registering the CST template from the Natbrainlab (http://www.natbrainlab.co.uk/atlas-maps) [20] into the T1-weighted images. Images were visually checked at each step for quality control of the image registration. The weighted overlap was introduced with consideration of the narrowing of the CST as it descends from the motor cortex to the PLIC. The weighted factor in each slice was calculated by multiplying the lesion-tract overlap on each slice by the ratio of the maximum cross-sectional area of the tract to the cross-sectional area of the tract on that particular slice. For a particular slice, *z*, containing *n*(*z*, *I*) voxels with intensity ≥ *I*, the weighted factor *f*(*z*, *I*) was calculated as

(1) $$\begin{array}{c} f\left(z,I\right)=\frac{n\left(z^{*},I\right)}{n\left(z,I\right)}, \end{array}$$

where *n*(*z*^∗^, *I*) indicates the number of voxels on slice *z*^∗^ containing the most voxels of intensity ≥ I. For each patient, the wLL volume was calculated using

(2) $$\begin{array}{c} V_{\text{weighted}}=\overset{m_{\max}}{\underset{m=1}{\sum }}\left(V_{\text{raw}}∙f\left(z\left(m\right),I\left(m\right)\right)\right., \end{array}$$

where *m*_max_ is the total number of intersecting voxels between the lesion map and CST map, *I*(*m*) is the intensity of the *m*^th^ voxel located on slice *z*(*m*), and *f*(*z*(*m*), *I*(*m*) is the weighting factor for the voxel.

### 2.5. FAA Estimation of CST and PLIC

The CST (or PLIC) fractional anisotropy asymmetry (FAA) is defined as the mean rFA between the affected (FAaff) and the unaffected (FAunaff) CST (or PLIC): rFA = FAunaff/FAaff. The FAAI was computed as a ratio: FAAI = (FAunaff − FAaff)/(FAunaff + FAaff) [21]. Before calculating the FAA of each patient's CST and PLIC, the diffusion MRI data were preprocessed in TORTOISE to correct for motion, eddy current, and geometric distortion [22, 23], and the diffusion data (the B0 image) were registered into respective T1-weighted images. We then estimated the diffusion tensor using a nonlinear least squares method in TORTOISE and generated a map of FA values.

The CST determined by calculating the weighted lesion-CST overlap was also used here. The MNI152 T1 template with 2 mm resolution was nonlinearly registered into patients' T1-weighted images, and the generated 3D deformation field was used on the PLIC template from JHU (http://neurovault.org/) to transform it to each patient's T1-weighted image space. Finally, the FAA estimations of CST and PLIC were calculated in MATLAB using a homemade script.

### 2.6. Statistical Analysis

The primary outcome was the UE-FM score 3 months after the stroke (3M UE-FM), and the secondary outcome was the UE-FM recovery percentage (UE-FM Pct). The mean and standard deviation (SD) were used to express normally distributed data; the median (P25–P75) and interquartile range were used to express nonnormally distributed data. Student's *t*-test and analysis of variance were used for normally distributed variables; the Mann-Whitney *U* test was used for asymmetrically distributed variables. Normally distributed variables were analyzed via Pearson correlation, and all others were analyzed via Spearman's rank correlation. Univariate and multivariate regression analyses were conducted to assess the factors influencing 3M UE-FM or UE-FM Pct. Feng et al. [16] defined UE‐FM scores ≤ 25 at 3 months as poor motor outcomes. The UE-FM recovery percentage was defined as UE − FM Pct = [(3M UE‐FM)–(baseline UE‐FM)]/[66 − (baseline UE‐FM)] [24]. Receiver operating characteristic (ROC) curve analysis was used to evaluate the cut-off point for CST-wLL on the baseline with the greatest sensitivity and specificity for predicting UE-FM 3 months poststroke and the UE-FM recovery percentage. The DeLong test was used to estimate the difference between ROC models. Statistical analyses were conducted in SPSS for Windows version 25.0 (SPSS Inc., Chicago, IL, USA) and software R (version 4.0.5). Statistical significance was defined as *p* < 0.05.

## 3. Results

### 3.1. Patient Characteristics

Twenty-eight patients completed the imaging and clinical assessments at both the baseline and 3-month follow-up (Figure 1).  Table 1 summarizes the clinical characteristics and baseline assessments. Patients' ages ranged from 46 to 83 years. Patients were assessed at 7–29 days after stroke onset and reassessed at 90–191 days. MRIs were scanned 10–31 days after onset. From a possible high score of 66, the baseline UE-FM scores ranged from 2 to 56, and the UE-FM scores 3 months poststroke ranged from 8 to 65.

### 3.2. MRI Statistics

We analyzed the sMRI-based lesion size (lesion volume), LL, and CST-wLL (cc) and the DTI-based PLIC-rFA, CST-rFA, PLIC-FAAI, and CST-FAAI. The normality test results showed that the LL, CST -wLL, PLIC-rFA, CST-rFA, PLIC-FAAI, and CST-FAAI were normally distributed, while lesion size was not. The log-transformed lesion size (log-lesion size) was normally distributed (Table 2, Supplementary Figure 1).

### 3.3. Correlation and Regression Analyses

Pearson correlation analysis was conducted for LL, CST-wLL, PLIC-rFA, PLIC-FAAI, CST-rFA, CST-FAAI, 3M UE-FM, and UE-FM Pct. Lesion size was log-transformed for the analysis. Log-lesion size, LL, CST-wLL, PLIC-rFA, PLIC-FAAI, CST-rFA, and CST-FAAI were all well correlated with 3M UE-FM and UE-FM Pct (Table 3). When age, gender, and education years were controlled, the partial correlation analysis showed a similar result (Supplementary Table 1).

**Figure 2:**
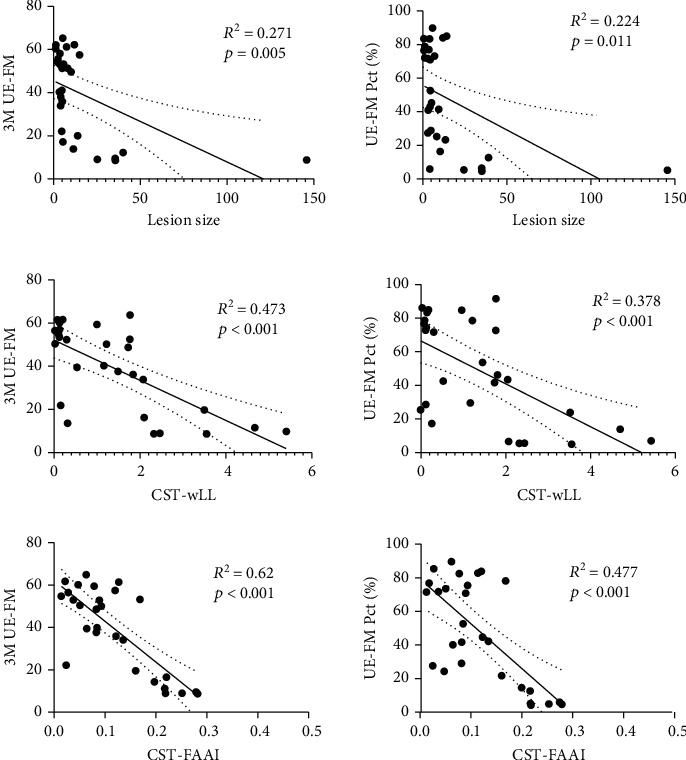
Univariate regression analysis for lesion size, CST-wLL, and CST-FAAI.

Univariate regression analysis was performed to evaluate whether the variance (*R*^2^) of the 3M UE-FM or UE-FM Pct could be explained by lesion size, LL, CST-wLL, PLIC-rFA, PLIC-FAAI, CST-rFA, or CST-FAAI (Table 4, Supplementary Figure 2). CST-wLL was more strongly correlated with 3M UE-FM than was lesion size. The CST-FAAI from the template mask also correlated significantly with 3M UE-FM (Figure 2). CST-wLL was more highly correlated with UE-FM Pct than was lesion size. The CST-FAAI from the template mask was also significantly correlated with UE-FM Pct (Figure 2). When multivariate regression analysis was performed with 3M UE-FM as the dependent variable and lesion size, LL, CST-wLL, PLIC-rFA, CST-rFA, PLIC-FAAI, and CST-FAAI as the independent variables, the model selection results based on stepwise showed that CST-FAAI was the most significant predictor for 3M UE-FM (adjusted *R*^2^ = 0.606, F = 42.484, beta = −0.788, *p* < 0.001). When multivariate regression analysis was performed with UE-FM Pct as the dependent variable and lesion size, LL, CST-wLL, PLIC-rFA, CST-rFA, PLIC-FAAI, and CST-FAAI as the independent variables, the model selection results based on stepwise showed that CST-FAAI was the most significant predictor for UE-FM Pct (adjusted *R*^2^ = 0.457, F = 23.734, beta = −0.691, *p* < 0.001).

### 3.4. CST-wLL Threshold Analysis

**Figure 3:**
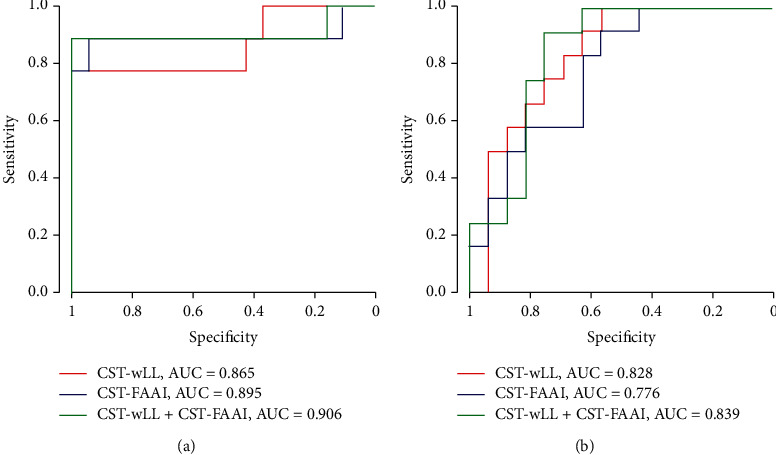
ROC for (a) poor motor outcomes (defined as 3M UE‐FM ≤ 25) and (b) considerable proportional recovery (defined as UE‐FM Pct ≥ 70%).

Consistent with Lin et al. [17], we defined the upper limb motor function outcome as poor if the UE-FM score was ≤25 at 3 months poststroke. Analysis of the ROC curve showed that when CST-wLL was used to predict motor outcome 3 months poststroke, the cut-off point was 2.068, with 77.8% sensitivity, 100% specificity, 100% PPV, 90.5% NPV, and 0.865 AUC (Figure 3). Hence, a CST‐wLL ≥ 2.068 cc on an MRI within 1 month indicated a poor function outcome. The CST-FAAI also showed a high predictive value, with an AUC of 0.895 (Table 5). The CST-wLL showed a comparable AUC from other classification models with CST-FAAI (*p* = 0.71, by DeLong test), and other metrics, including sensitivity, specificity PPV, and NPV, changed only slightly. Including extra-CST-FAAI did not significantly increase the AUC (*p* = 0.58, CST-wLL vs. CST-wLL and CST-FAAI, by DeLong test).

Prabhakaran et al. [24] discovered that some patients with stroke had a 70% near-fixed proportional upper limb motor recovery within 3 months. Analysis of the ROC curve showed that when CST-wLL was used to predict the motor recovery percentage, the cut-off point was 1.799, with 100% sensitivity, 56.2% specificity, 63.2% PPV, 100% NPV, and 0.828 AUC (Figure 3). In other words, a CST‐wLL < 1.799 cc within 1 month indicated a considerable proportional recovery (≥70%) of the patient's upper limb motor function within 3 months after onset. The CST-FAAI showed lower accuracy, sensitivity, specificity, PPV, NPV, and AUC (Table 6).

## 4. Discussion

We found that CST-wLL obtained from routine sMRI examinations within 1 month of stroke onset was closely correlated with upper limb motor function outcomes 3 months poststroke. CST-wLL was more relevant than lesion size as a predictor of upper limb motor recovery. CST‐wLL ≥ 2.068 cc indicated serious CST damage and a poor outcome (100%). CST‐wLL < 1.799 cc within 1 month poststroke indicated that patients would recover a considerable proportion (≥70%) of their upper limb motor functions within 3 months after stroke onset. CST-FAAI may be the optimal predictor for upper limb motor outcomes in patients after stroke. However, CST-wLL showed a comparable AUC to that of DTI-derived metrics, such as CST-FAAI, for predicting recovery of upper limb motor function and proportional recovery.

As a predictor of upper limb motor function prognosis 3 months poststroke, CST-wLL can be used as a predictive imaging biomarker to classify patients for rehabilitation. This would help practitioners set more realistic rehabilitation goals, integrate resources, and improve efficiency. A CST‐wLL ≥ 2.068 cc indicated a poor outcome (100%), and these patients could receive specific initial rehabilitation based on predictive stratification [17, 25]. A recent study suggested that a 3-week CST-wLL was the strongest predictor of the ability to grasp and control finger forces 6 months poststroke [26]. A CST‐wLL > 5.5 cc strongly predicted low-to-minimal recovery in unimanual motor impairment and bimanual activity performance (specificity: 0.91) [27].

In our study, CST-FAAI may be the optimal predictor for upper limb motor outcomes. However, CST-wLL showed a comparable precise prediction with DTI-derived metrics such as CST-FAAI. The rFA and FAAI between ipsilateral and contralateral CST are the most commonly used DTI-derived metrics for predicting motor recovery [28]. Higher baseline FA and rFA values have been correlated with better motor recovery and can predict motor function outcomes in patients after ischemic stroke [14]. A meta-analysis including fifteen studies with 414 patients revealed that FA in the subacute phase after ischemic stroke is a good predictor of functional motor recovery and showed moderate quality based on the GRADE system [29]. However, these DTI-derived metrics represent diffusion directions of the water molecules and their patterns along the axon, i.e., “CST structural characteristics.” Calculating the CST-wLL enables quantifying how much “CST structural integrity” has been damaged due to stroke; this allows more accurately predicting the recovery of upper limb motor function [26]. Few studies have compared the performance of different neuroimaging modalities. Doughty et al. [30] suggested that CST lesion load in the acute phase predicts 3-month outcomes better than the FAA of regions of interests (ROIs) distal to the lesion. A recent study estimating CST injury by the proposed method with diffusion metrics extracted from the diffusional kurtosis imaging (DKI) sequence and with the first principal component (PC1) of the metrics found that DKI_AK, AFD_total, and PC1 showed similar predictive values to those of wLL for functional outcomes [31]. Although previous studies have confirmed that FA is a good predictor of 3-month functional outcomes, our results suggested that the AUCs for CST-wLL in the subacute phase were similar to those of the FAAI. Another microstructural study suggested that the baseline asymmetry measures in the PLIC for the orientation dispersion index of the neurite orientation dispersion and density imaging (NODDI) model explained 83% of the variance of the upper extremity FM outcomes whereas FA values explained only 49% [10]. However, the NODDI model is not routinely available in stroke units. The method proposed in our study for estimating CST injury is more easily implemented in clinical settings.

As a potential imaging biomarker for poststroke motor outcomes, wLL has some advantages over functional MR and DTI, including high patient compliance and examination convenience [25, 32]. T2-WI sequences are routinely available for patient care, whereas fMRI or DTI is not a routine examination in stroke units and rehabilitation units considering its costs and hardware requirements, especially in developing countries. We used the CST model from the standard template [15] instead of the tractography results from locally acquired DTI data of age-matched healthy controls. As the major purpose of this study is to compare between the prediction performance with structural MRI only and with DTI, it is important to not acquire any new DTI data for the calculations within the structural MRI group. In this study, PLIC-rFA, PLIC-FAAI, CST-rFA, and CST-FAAI from the CST template were highly correlated with the outcomes of upper limb motor function, which is consistent with the results of previous studies [15]. Prior studies that calculated CST-wLL used heterogeneous methods, and the templates used and methods of overlap varied substantially [16, 26, 33]. However, these studies showed significant agreement between methods for estimating CST injury, suggesting that these methods are relatively precise [34].

**Figure 4:**
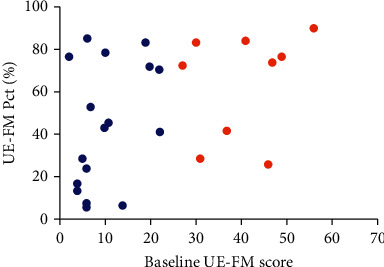
For patients with serious initial dysfunction (blue points), the recovery percentage varied from limited to considerable. This was the same for patients with mild initial dysfunction (orange points).

CST-wLL was also highly correlated with the proportion of recovery of upper limb motor function. Previous studies [24, 35] found that approximately 70% of the maximum recovery potential of most stroke patients, apart from those with serious initial motor impairment, may be realized. Our results showed that the initial severity of the motor impairment did not predict the proportion of recovery. Some patients with serious initial dysfunction still achieved a substantial proportion (≥70%) of functional recovery, while some patients with mild initial dysfunction showed limited recovery potential (Figure 4). CST-wLL showed considerable predictive values for proportional recovery. Our results suggested that CST-wLL showed considerable predictive values for proportional recovery. CST‐wLL < 1.799 cc within 1 month indicated recovery of a large proportion (≥70%) of upper limb motor function within 3 months after onset. This was consistent with the results of a previous study [16], which showed that the CST-wLL could be used to predict the recovery proportion. Stinear et al. [21] indicated that an FAAI of 0.25 is a “point of no return,” beyond which functional potential is severely limited. In this study, CST-wLL showed higher accuracy, sensitivity, specificity, PPV, NPV, and AUC than did FAAI. Consequently, CST-wLL can be used as a classification variable to predict recovery potential for individual stroke patients [36], thus directing clinical motor function rehabilitation and increasing rehabilitation efficiency.

Our study and methodology have some limitations. First, although we improved spatial standardization, individual differences must be considered when taking the CST localization from the standardized mask, especially in brains with large lesions, where the results may be less accurate than those in brains with small lesions. Second, the study only included patients with first-onset acute ischemic stroke and excluded patients with second- or third-onset stroke, which may affect the generalizability of the results. Additionally, the sample size was too small to incorporate rehabilitation treatment factors, drug treatment factors, depression, and perfusion therapy for multiple regression analysis to further clarify the weight of CST-wLL as a neuroimaging biomarker in predicting upper limb motor recovery. More pioneering approaches, such as machine learning algorithms and deep learning for classification and prediction, have been proposed as they can capture complex and nonlinear relationships [37] and show great potential in integration of voluminous clinical data and imaging data for predicting motor function prognosis at much quicker speeds and at higher accuracy without bias [38–41]. However, machine learning algorithms generally require a large sample size and diverse sample distribution to train the algorithm and improve the generalization, and it remains challenging to implement these algorithms in clinical routines [42]. The current study provides a practical way to predict the motor outcomes in the clinical environment, and the prediction model can be further improved by combining with advanced machine learning algorithms as we have a larger sample size from different hospitals in the future. Finally, as a threshold for classification of upper limb motor outcomes and recovery potential, CST-wLL must be further defined and verified with larger sample sizes in future studies.

CST-wLL obtained from routine sMRI within 1 month after stroke onset may serve as a potential imaging biomarker for predicting upper limb motor function prognosis and recovery potential 3 months poststroke. This study optimized the prediction model (protocol) in the clinical environment by comparing the performance of sMRI-derived CST-wLL and DTI-derived metrics for predicting upper limb motor recovery. Including extra-DTI will not induce significant benefits. However, as a clinical predictive imaging biomarker of motor function recovery and a classification variable to guide future stroke rehabilitation, CST-wLL still requires verification in studies with larger samples. Accurate metrics and predictive models are critical for defining an optimal neurorehabilitation protocol that will promote motor recovery and maximize functional outcomes for stroke survivors.

## Figures and Tables

**Table 1:** Patient characteristics. *N* (%) for categorical variables; mean ± SD for continuous variables. UE-FM outcome groups: severe: 3M UE-FM score, ≤25; mild-moderate: 3M UE-FM score, 26–66. *p* value: the statistical difference between two groups of patients.

Variable	All (*n* = 28)	UE-FM outcome groups		
		Severe (*n* = 9)	Mild-moderate (*n* = 19)	*p* value
Sex				
Male	19 (67.9%)	8 (88.9%)	11 (57.9%)	0.20
Female	9 (32.1%)	1 (11.1%)	8 (42.1%)	
Age (years)	62.8 ± 9.7	60.3 ± 10.2	63.9 ± 9.5	0.39
Education (years)	8 ± 2.9	9 ± 3.7	7.6 ± 2.5	0.32
Days of baseline MRI	19.8 ± 6.1	21 ± 6.3	19.2 ± 6.1	0.49
Days of baseline UE-FM	16.6 ± 6.8	17.7 ± 7.1	16.2 ± 6.8	0.60
Days of 3M UE-FM	112 ± 16.4	114.9 ± 20.5	110.7 ± 14.5	0.59
Baseline UE-FM score	19.6 ± 16.4	6.3 ± 3.0	25.9 ± 16.4	<0.001
3M UE-FM score	39.4 ± 19.9	13.6 ± 5	51.6 ± 9.5	<0.001
FM Pct (%)	47.5 ± 30.5	12 ± 8.7	64.3 ± 20.8	<0.001

**Table 2:** MRI statistics. Mean ± SD and median (P25, P75) for continuous variables. UE-FM outcome groups: severe: 3M UE-FM score, ≤25; mild-moderate, 3M UE-FM score, 26–66. *p* value: the statistical difference between two groups of patients. Log-lesion size: log-transformed lesion size.

Variable	All (*n* = 28)	UE-FM outcome groups		
		Severe (*n* = 9)	Mild-moderate (*n* = 19)	*p* value
Lesion size (cc)	4.906 (3.539, 11.732)	24.080 (10.052, 34.955)	3.964 (2.853, 6.213)	0.002
Log-lesion size	0.803 ± 0.521	1.286 ± 0.503	0.575 ± 0.351	0.002
LL (cc)	1.372 ± 0.947	2.128 ± 0.996	1.013 ± 0.695	0.011
CST-wLL (cc)	1.431 ± 1.464	2.714 ± 1.789	0.823 ± 0.760	0.013
PLIC-rFA	0.747 ± 0.158	0.592 ± 0.139	0.820 ± 0.106	0.001
PLIC-FAAI	0.155 ± 0.111	0.265 ± 0.108	0.103 ± 0.065	0.002
CST-rFA	0.795 ± 0.126	0.666 ± 0.119	0.856 ± 0.072	0.001
CST-FAAI	0.119 ± 0.081	0.205 ± 0.078	0.079 ± 0.042	0.001

**Table 3:** Correlation analysis. ^∗∗^*p* < 0.001, ^∗^*p* < 0.05.

	3M UE-FM	UE-FM Pct
Log-lesion size	−0.67^∗∗^	−0.645^∗∗^
LL	−0.623^∗∗^	−0.614^∗^
CST-wLL	−0.688^∗∗^	−0.615^∗∗^
PLIC-rFA	0.739^∗∗^	0.62^∗∗^
PLIC-FAAI	−0.739^∗∗^	−0.628^∗∗^
CST-rFA	0.774^∗∗^	0.677^∗∗^
CST-FAAI	−0.788^∗∗^	−0.691^∗∗^

**Table 4:** Univariate regression analysis.

	3M UE-FM		UE-FM Pct	
	R^2^	*p* value	R^2^	*p* value
Lesion size	0.271	0.005	0.224	0.011
LL	0.388	<0.001	0.377	<0.001
CST-wLL	0.473	<0.001	0.378	<0.001
PLIC-rFA	0.546	<0.001	0.384	<0.001
PLIC-FAAI	0.546	<0.001	0.394	<0.001
CST-rFA	0.599	<0.001	0.458	<0.001
CST-FAAI	0.62	<0.001	0.477	<0.001

**Table 5:** ROC analyses for predicting 3M UE-FM.

Predictor variables	Accuracy	Sensitivity	Specificity	PPV	NPV	AUC
Lesion size	0.857	0.778	0.895	0.778	0.895	0.871
LL	0.786	0.889	0.737	0.615	0.933	0.836
wLL	0.929	0.778	1	1	0.905	0.865
PLIC-rFA	0.786	1	0.684	0.6	1	0.912
PLIC-FAAI	0.786	1	0.684	0.6	1	0.912
CST-rFA	0.929	0.889	0.947	0.889	0.947	0.895
CST-FAAI	0.929	0.889	0.947	0.889	0.947	0.895
CST-wLL & CST-FAAI	0.964	0.889	1	1	0.95	0.906

**Table 6:** ROC analyses for predicting UE-FM Pct.

Predictor variables	Accuracy	Sensitivity	Specificity	PPV	NPV	AUC
Lesion size	0.786	0.5	1	1	0.727	0.766
LL	0.821	0.917	0.75	0.733	0.923	0.818
wLL	0.75	1	0.562	0.632	1	0.828
PLIC-rFA	0.821	0.833	0.812	0.769	0.867	0.818
PLIC-FAAI	0.821	0.833	0.812	0.769	0.867	0.818
CST-rFA	0.714	0.917	0.562	0.611	0.9	0.776
CST-FAAI	0.714	0.917	0.562	0.611	0.9	0.776

## Data Availability

All data included in this study are available upon request by contact with the corresponding author.

## Glossary

CST: Corticospinal tract
MRI: Magnetic resonance imaging
sMRI: Structural magnetic resonance imaging
DTI: Diffusion tensor imaging
UE: Upper extremity
UE-FM: Upper extremity module of the Fugl-Meyer assessment
3M UE-FM: The UE-FM score 3 months after the stroke
UE-FM Pct: UE-FM recovery percentage
LL: Lesion load
CST-wLL: CST-weighted lesion load
PLIC: Posterior limb of the internal capsule
FAA: Fractional anisotropy asymmetry
FAAI: Fractional anisotropy asymmetry index
rFA: Ratio of fractional anisotropy
AUC: Area under the curve
PPV: Positive predictive value
NPV: Negative predictive value
fMRI: Functional magnetic resonance imaging
TOAST: Trial of ORG 10172 in Acute Stroke Treatment
FLAIR: Fluid-attenuated inversion recovery
ROC: Receiver operating characteristic
ROIs: Regions of interests
GRADE: Grading of Recommendations Assessment, Development and Evaluation
DKI: Diffusional kurtosis imaging
NODDI: Neurite orientation dispersion and density imaging.

## Supplementary Materials

Supplementary Materials Supplementary Figure 1. Statistical differences in MRI between severe and mild-moderate patients. ^∗^*p* < 0.05. Supplementary 2. Univariate regression analysis. ^∗^Removed outlier point (lesion size = 145.48).Supplementary Table 1.Partial correlation analysis. Note: ^∗∗^*p* < 0.001 and ^∗^*p* < 0.05. Supplementary Table 2. Differences in brain images between severe and mild-moderate patients. Red ROI: lesion mask; blue ROI: CST mask; red circle: PLIC mask; yellow circle: CST mask.
