# Supplementary material for: Performance Comparison of Different Neuroimaging Methods for Predicting Upper Limb Motor Outcomes in Patients after Stroke
Source: Neural Plast. 2022 Jun 6;2022:4203698. doi: 10.1155/2022/4203698 (PMC9192322; doi:10.1155/2022/4203698)
Supplement: Supplementary Materials — Supplementary Figure 1. Statistical differences in MRI between severe and mild-moderate patients. ∗p < 0.05. Supplementary 2. Univariate regression analysis. ∗Removed outlier point (lesion size = 145.48).Supplementary Table 1.Partial correlation analysis. Note: ∗∗p < 0.001 and ∗p < 0.05. Supplementary Table 2. Differences in brain images between severe and mild-moderate patients. Red ROI: lesion mask; blue ROI: CST mask; red circle: PLIC mask; yellow circle: CST mask. [file 4203698.f1.zip › Supplementary Table 1.docx]

Supplementary Table 1. Partial Correlation Analysis. Note: **: *p* < 0.001; *: *p* < 0.05.

|  | 3M UE-FM | UE-FM Pct |
| --- | --- | --- |
| Log-lesion size | −0.665** | −0.645* |
| LL | −0.641* | -0.649** |
| CST-wLL | -0.676** | -0.652** |
| PLIC-rFA | 0.763** | 0.683** |
| PLIC-FAAI | -0.775** | -0.704** |
| CST-rFA | 0.753** | 0.7** |
| CST-FAAI | -0.77** | -0.715** |
