# Supplementary material for: Performance Comparison of Different Neuroimaging Methods for Predicting Upper Limb Motor Outcomes in Patients after Stroke
Source: Neural Plast. 2022 Jun 6;2022:4203698. doi: 10.1155/2022/4203698 (PMC9192322; doi:10.1155/2022/4203698)
Supplement: Supplementary Materials — Supplementary Figure 1. Statistical differences in MRI between severe and mild-moderate patients. ∗p < 0.05. Supplementary 2. Univariate regression analysis. ∗Removed outlier point (lesion size = 145.48).Supplementary Table 1.Partial correlation analysis. Note: ∗∗p < 0.001 and ∗p < 0.05. Supplementary Table 2. Differences in brain images between severe and mild-moderate patients. Red ROI: lesion mask; blue ROI: CST mask; red circle: PLIC mask; yellow circle: CST mask. [file 4203698.f1.zip › Supplementary Table 2.docx]

Supplementary Table 2. Differences in Brain images between severe and mild-moderate patients. Red ROI: lesion mask. Blue ROI: CST mask. Red circle: PLIC mask. Yellow circle: CST mask.

| Case A from Severe Group | Case B from Mild- moderate Group |
| --- | --- |
| Lesion size (cc) = 4.524 | Lesion size (cc) = 14.0464 |
| CST-wLL (cc) = 2.082  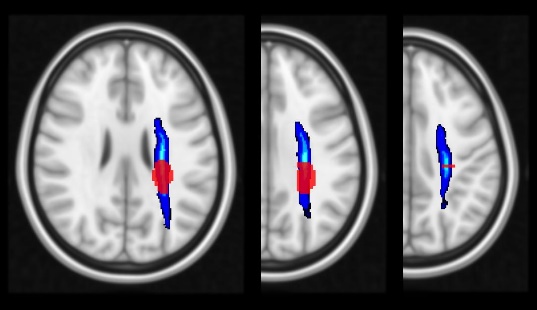 | CST-wLL (cc) = 0.032  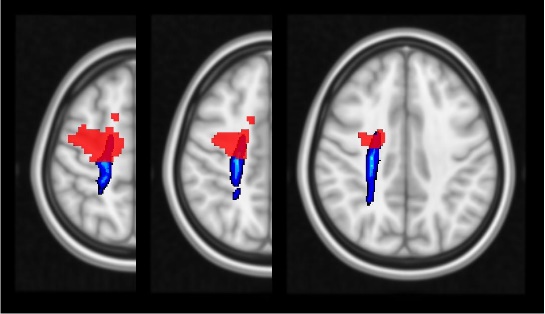 |
| PLIC-rFA = 0.562 PLIC-FAAI = 0.28  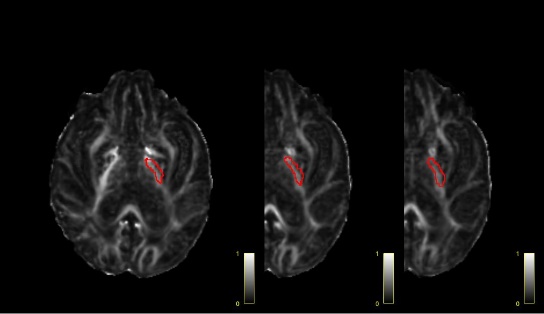 | PLIC-rFA = 0.945 PLIC-FAAI = 0.028  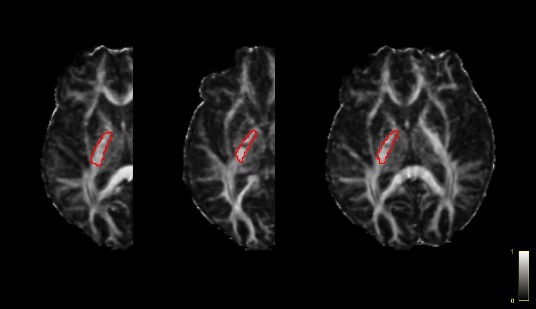 |
| CST-rFA = 0.64 CST-FAAI = 0.219  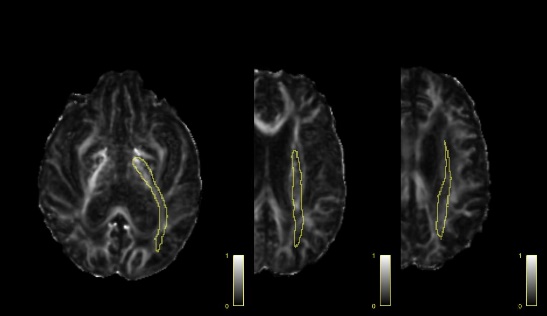 | CST-rFA = 0.95 CST-FAAI = 0.026  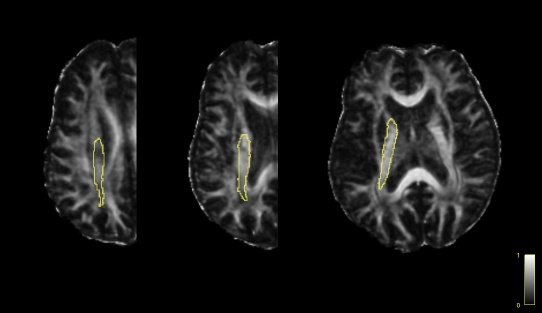 |
