# Supplementary figures and images for: Performance Comparison of Different Neuroimaging Methods for Predicting Upper Limb Motor Outcomes in Patients after Stroke
Source: Neural Plast. 2022 Jun 6;2022:4203698. doi: 10.1155/2022/4203698 (PMC9192322; doi:10.1155/2022/4203698)

Supplementary Figure 2. Univariate regression analysis. *: Removed the outlier point (lesion size=145.48).


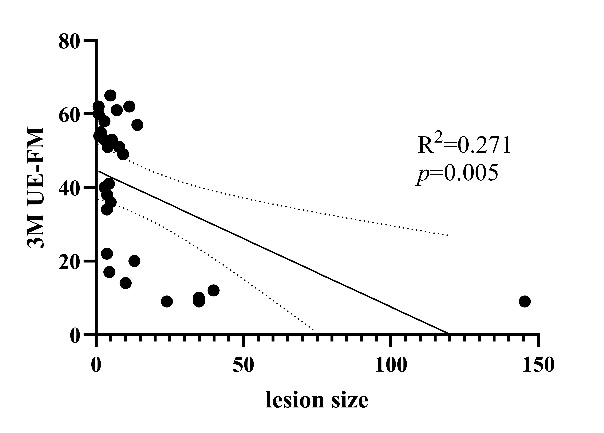

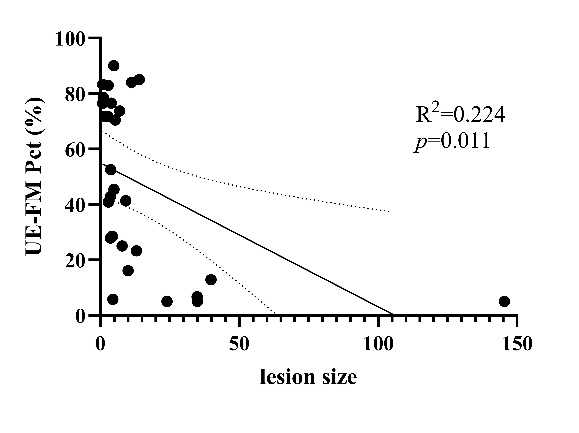

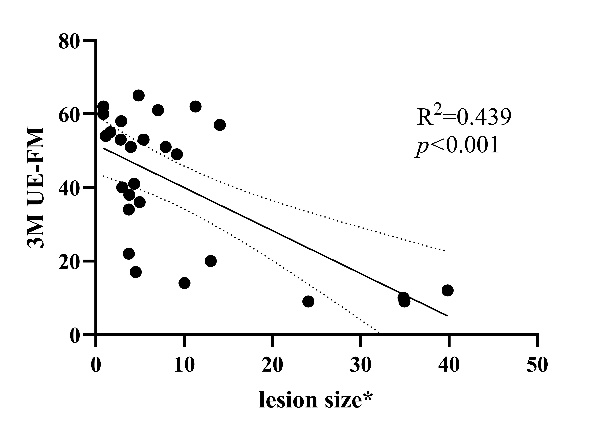

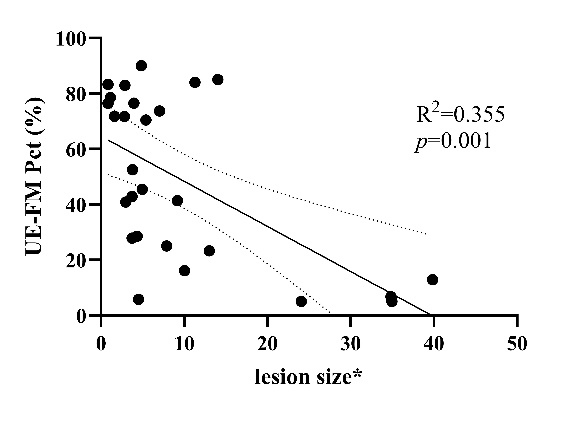

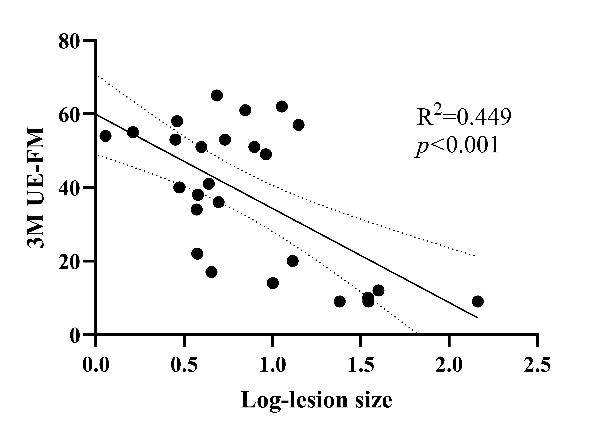

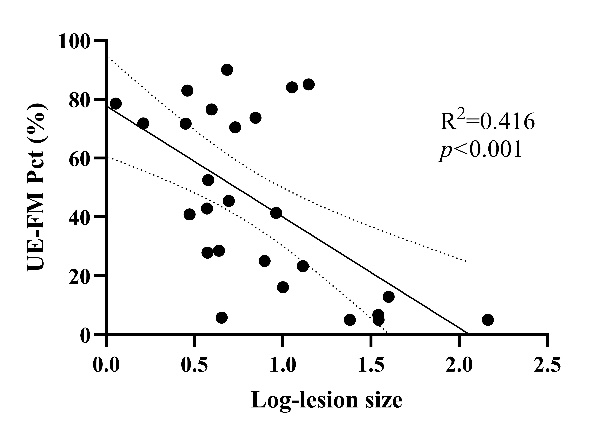

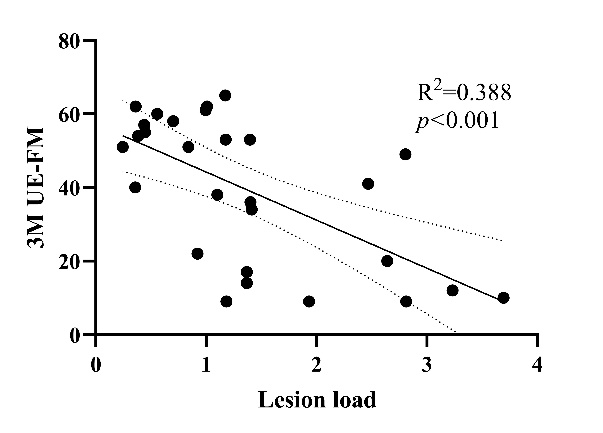

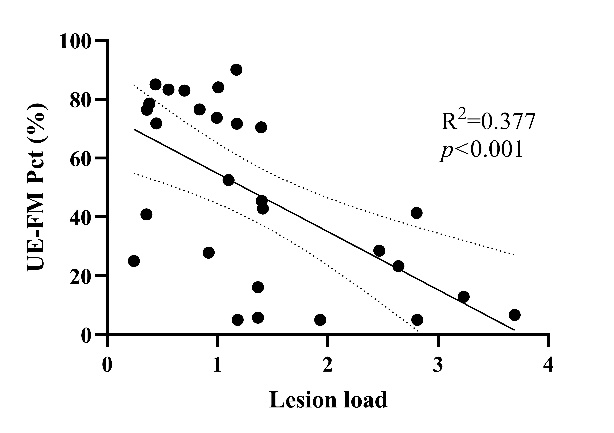

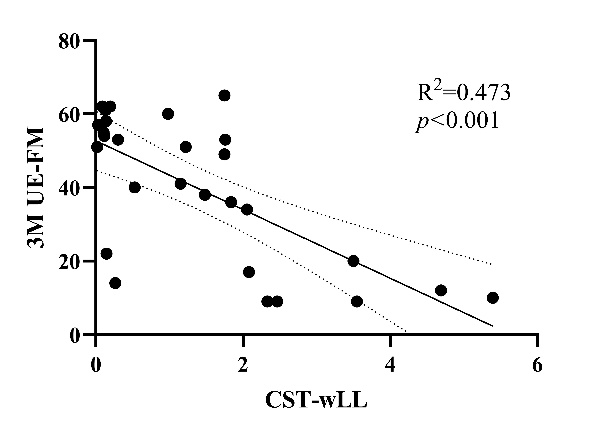

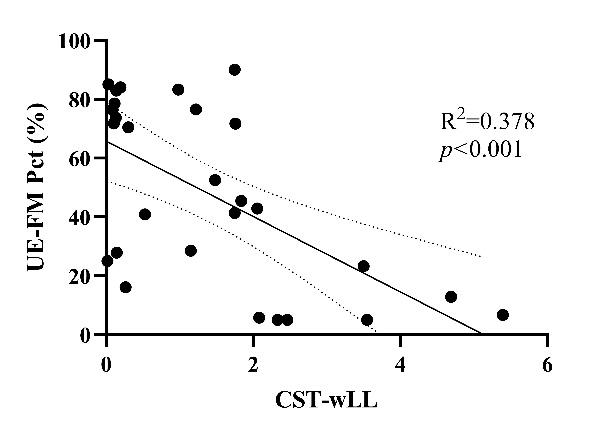

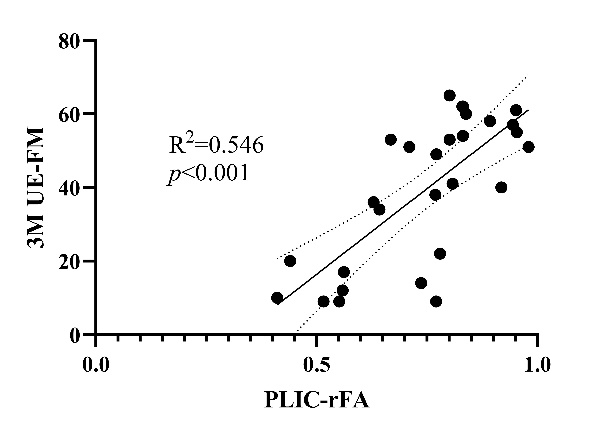

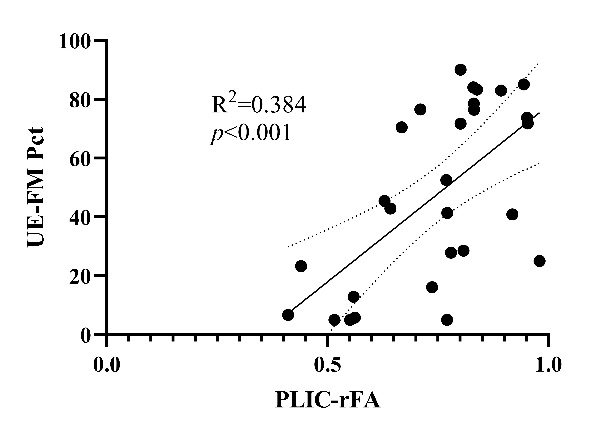

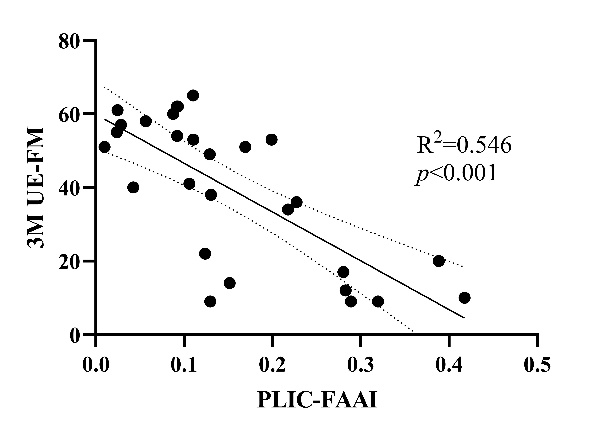

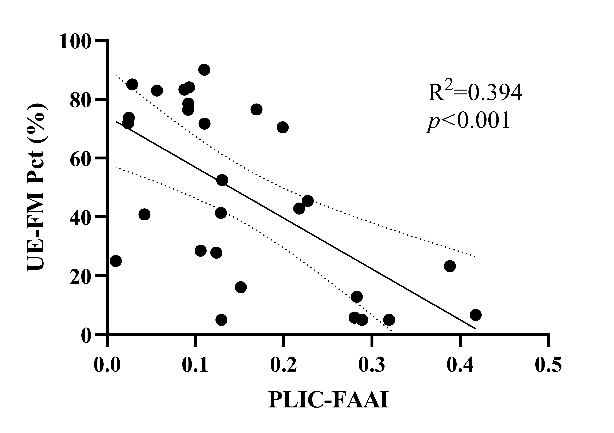

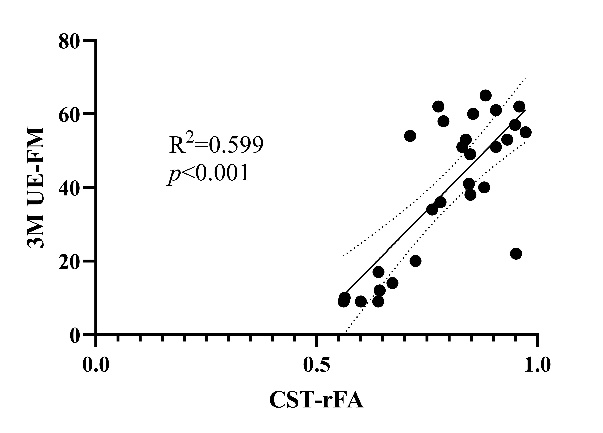

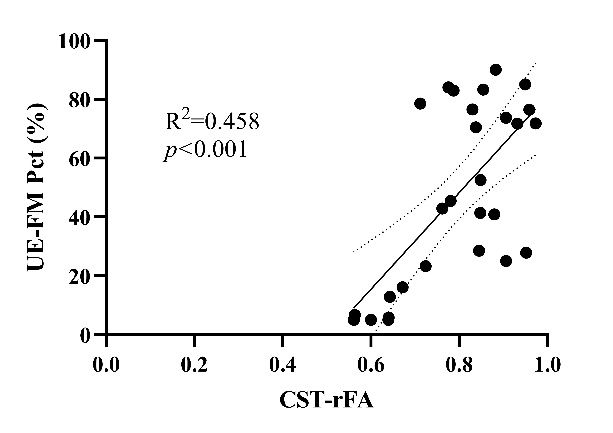

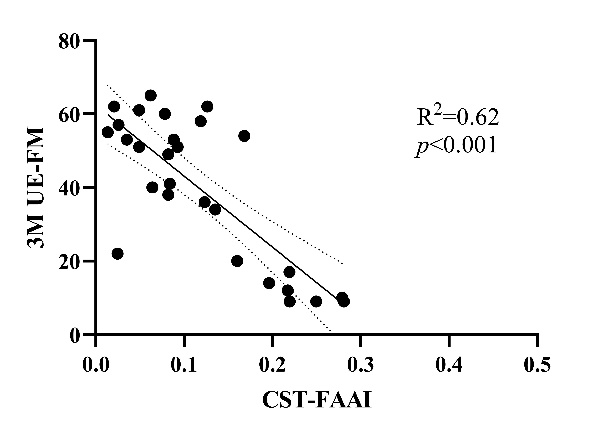

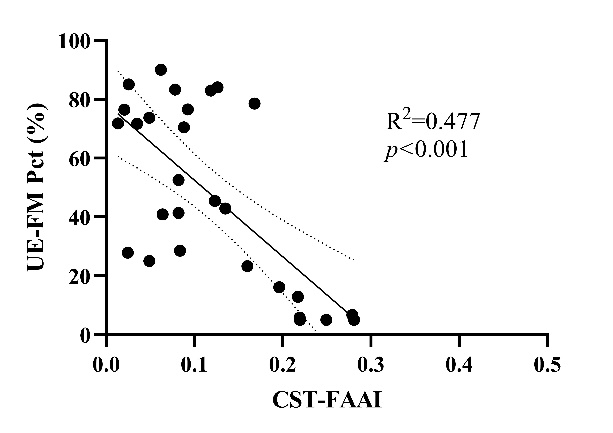

Supplement: Supplementary Materials — Supplementary Figure 1. Statistical differences in MRI between severe and mild-moderate patients. ∗p < 0.05. Supplementary 2. Univariate regression analysis. ∗Removed outlier point (lesion size = 145.48).Supplementary Table 1.Partial correlation analysis. Note: ∗∗p < 0.001 and ∗p < 0.05. Supplementary Table 2. Differences in brain images between severe and mild-moderate patients. Red ROI: lesion mask; blue ROI: CST mask; red circle: PLIC mask; yellow circle: CST mask. [file 4203698.f1.zip › Supplementary Figure 2.docx]
